# HIV-Positive Youth's Perspectives on the Internet and eHealth

**DOI:** 10.2196/jmir.6.3.e32

**Published:** 2004-09-29

**Authors:** Sarah Flicker, Eudice Goldberg, Stanley Read, Tiffany Veinot, Alex McClelland, Paul Saulnier, Harvey Skinner

**Affiliations:** ^5^Positive Youth OutreachAIDS Committee of TorontoToronto ONCanada; ^4^Canadian AIDS Treatment Information ExchangeToronto ONCanada; ^3^Faculty of MedicineUniversity of TorontoToronto ONCanada; ^2^Department of PaediatricsThe Hospital for Sick ChildrenToronto ONCanada; ^1^Department of Public Health SciencesUniversity of TorontoToronto ONCanada

**Keywords:** Youth, HIV, Internet, health promotion

## Abstract

**Background:**

Globally, half of all new HIV infections occur among young people. Despite this incidence, there is a profound lack of resources for HIV-positive youth.

**Objective:**

To investigate Internet access, use and acceptability as a means for health promotion and health service delivery among HIV-positive youth.

**Methods:**

A community-based participatory approach was used to conduct a mixed methods research study. Thirty-five qualitative in-depth semi-structured interviews were conducted with youth (ages 12-24) living with HIV in Ontario. Also, brief structured demographic surveys were administered at the time of the interview. A stakeholder group of youth living with HIV, professionals and researchers collaboratively analyzed the data for emerging themes.

**Results:**

Five main themes were identified with respect to the youth's use of and interest in the Internet as a health promotion strategy. These include: (1) high rates of Internet use and access; (2) issues around public and private terminals; (3) their use of the Internet primarily for communication and entertainment; (4) the rarity of health information seeking behavior in this group; and (5) wanting "one-stop shopping" from an e-health site. HIV-positive youth were enthusiastic about the possibility of content that was developed specifically to target them and their needs. Also, they were keen about the possibilities for increased social support that youth-specific online chat rooms and message boards might provide.

**Conclusion:**

Given high rates of use, access and interest, the Internet provides an important way to reach young people living with HIV using health services and health promotion programs. The onus is on e-Health developers to understand the particular needs of HIV-positive youth and create relevant content.

## Introduction

Globally, half of all new HIV infections occur among young people [1]. Currently there are an estimated 11.8 million youth aged 15-24 years living with HIV/AIDS [1]. In Canada, youth, particularly young women aged 15-29, represent a growing population who are being infected with HIV and AIDS [2,3]. As of June 2002, 13279 youth and young adults under the age of 29 had tested positive for HIV in Canada [4]. Due to under-reporting and under-diagnosis, as well as a long asymptomatic period, the actual prevalence of HIV in youth is likely much higher than indicated in official statistics. Surveillance data in the United States shows that although AIDS incidence is declining, there has not been a comparable decline in the number of newly diagnosed HIV cases among youth [5].

Despite this prevalence and incidence, there is a profound lack of resources for HIV-positive youth [6]. In particular, youth-­accessible resources outlining treatment options are scarce. Although material is available to help adults make treatment decisions, many of these resources are not appropriate for youth because they fail to address their unique clinical and developmental challenges. Furthermore, texts written for adults are often intimidating to younger audiences both because of language and literacy barriers and the less engaging ways in which information has traditionally been presented.

While many HIV-positive adolescents are at early stages in the course of their disease, health promotion messages are very important for them. Studies of adolescents living with HIV have shown high morbidity and mortality rates [7,8]. Other studies looking at the subjective health experience have documented that a quarter of those interviewed described their health as "fair" or "poor." [9] These findings illustrate the importance of treatment, self-care and prevention of co-infections for this population.

### Treatment and Self-care Needs

HIV-positive youth are unique in their treatment and self-care needs. Many youth for whom antiretroviral medications are clinically indicated choose not take them [10]. Many do not access health care services. Youth may have perceptions of treatment that differ from adults [11], favoring a present quality of life over improving biological markers. In contrast to adults, peer influence has been identified as one of the key factors affecting youth treatment decision making [12,13]. Thus, there is a strong need for peer-driven resources about HIV/AIDS treatment, which are presented in youth-friendly formats. Moreover, these resources need to be sensitive to the ways in which self-care and treatment decisions are contextualized within the broader scope of these youth's lives.

Adolescence and early adulthood are the stages when lifelong health and social behavior patterns are formed. HIV-positive youth are particularly vulnerable during this period, as they experience disproportionate rates of: homelessness [14,15]; sexual and physical abuse [16,17]; financial difficulties [18,21]; addictions [22]; legal concerns [20,23]; social isolation and stigma [9,23]; and mental health concerns [16,24]. Often, the immediacy of these social and structural determinants of health may overshadow worries about HIV infection [20]. This results in a need for information that is sensitive to the unique situations of HIV-positive youth, while framing their experiences within the perspective of normal youth development to avoid further marginalization and stigmatization.

### Potential of the Internet

There is a growing literature that emphasizes the potential of the Internet, not only for health promotion [25,26], but also as a community development tool [27,28]. The Internet provides innovative ways of engaging youth, allowing opportunities to assess and address their needs and to provide them with a means of offering each other support. Research has demonstrated that computers can attract young adults to participate in health assessments and behavior change programs, in ways and numbers that are not possible using traditional approaches [29,30]. Internet technology can be easily updated, is available 24 hours per day, and enables self-directed learning. It can be reached by those in remote and isolated settings, facilitates repeat use and can be anonymously accessed. Finally, information presented online can be of a highly graphical, interactive nature, and thus be able to reach users who may not have age-appropriate literacy skills [30].

In 1994, only 17% of young people were estimated to be using the Internet. Data from 2000 however, suggest that between 92% and 99% of Canadian youth used the Internet regularly [31]. Indeed, the digital divide in Canada is narrowing. Among households with less than $20,000 incomes, 77% of youth reported regular Internet use in 2000. Given the rapid growth of Internet use, this number has probably grown considerably [31]. American youth are also online: 73% of 12-17 year olds in the US use the Internet regularly [32], and 95% of all teens have ever been online [33]. Generally, youth are more likely than their adult counterparts to use the Web and are 'early adopters' of technology [34].

Research with adult populations living with HIV has demonstrated that computer-based health services can improve a patient's quality of life and promote more efficient use of health care systems [35-37]. Furthermore, qualitative studies have shown that HIV-positive adults use the Internet for a wide variety of functions including communication, advocacy and commerce [38,39]. Kalichman found that individuals with HIV who used the Internet were more likely to be better informed about HIV treatment and self-care than those who did not [40,41]. However, he also found that there was a "digital divide": those accessing the Internet were more likely to be better educated and report higher incomes [40-41].

Despite the growing popularity of the Internet as a health information resource [43], little research has been conducted on the feasibility of using the Internet as a health promotion strategy with HIV-positive youth. One of the aims of this study was to investigate Internet access, use and acceptability among this vulnerable and marginalized population.

## Methods

A community-based participatory research model [44,45] was used to assess the needs of Canadian HIV-positive youth. A stakeholder group of HIV-positive youth (trained as community researchers) and supporting professionals collaboratively developed the research design, instruments and protocol. Qualitative methods were selected for their ability to explore issues 'in-depth' and allow participants to express their thoughts and feelings 'in their own words.' Thirty-five interviews were conducted with a diverse group of HIV-positive youth across Ontario. The interviews were semi-structured and probed around four main areas of interest: a) future goals; b) social support; c) treatment and self-care issues; and d) online interests and behaviors. In addition, brief structured surveys were administered at the conclusion of each interview. Surveys asked about demographics (e.g., age, sex, sexuality, etc.) and Internet use. This paper will focus on the online component (other findings have been reported elsewhere).

Using a maximum variation sampling scheme, a sampling frame was developed that ensured diversity in age, sex, sexuality, age of diagnosis, ethno-racial identity and geographic region [46]. Youth were recruited through AIDS-serving organizations, youth-serving organizations, hospitals, and health clinics. In some cases, youth workers and health care providers approached young people in their case load and told them about the study. In other cases, recruitment flyers were simply posted. Also, young people who had already participated were encouraged to tell other HIV-positive youth who they knew about the study (snowball recruitment).

In all cases, youth approached the research team directly. Participation was limited to youth who: a) were between the ages of 12 and 24 years; b) were identified as HIV-positive through self-report; c) had the ability to communicate in either English or French; and d) had lived in Ontario for the last three months. Each received a $20 honorarium for participation. Standard procedures were employed for obtaining informed consent (approved by the University of Toronto Human Subjects Ethical Review Committee and the Research Ethics Review Board at The Hospital for Sick Children). Two interviews were conducted in French; 33 interviews were conducted in English. Table 1 provides a breakdown of our final sample.

**Table 1:** Sample characteristics

**Characteristic**	**Number Interviewed(Percentage)**
**Gender**	
Male	22 (63%)
Female	13 (37%)
**Age**	
12-15	6 (17%)
16-19	12 (34%)
20-24	17 (49%)
**Sexuality**	
LGBTQ^1^	8 (23%)
Heterosexual	27 (77%)
**Diagnosis**	
Last 12 months	15 (43%)
Longer than 12 months	13 (37%)
Perinatal	7 (20%)
**History of Street Involvement**	
Yes	24 (68%)
No	11 (31%)
**Ethno-Racial Identity**	
White, European, Canadian	19 (54%)
African/Caribbean	10 (29%)
First Nation/Aboriginal	3 (9%)
Unknown/Other^2^	3 (9%)
**Geographic Location**	
Large Urban	28 (80%)
Small Urban/Rural	4 (11%)
Northern	3 (9%)
^1^ Lesbian, Gay, Bisexual, Transsexual, Queer or Questioning ^2^ Unspecified, Chinese, South-East Asian	

Interviews lasted between 35 and 95 minutes. Generally, they were taped and transcribed verbatim. In one case, a youth did not want to be audio-taped and copious notes were taken during the interview. In another case, a youth wanted to write out his own answers rather than talking into a tape-recorder. At the conclusion of each interview, youth were asked to fill out a brief demographic survey and invited to continue to participate in the research project. In addition, they were provided with a list of youth-friendly health and service agencies in their area.

A modified grounded theory interpretive approach guided the analyses [47-49]. A sub-sample of 10 transcripts, stripped of identifying names and places, were returned to the stakeholder group of HIV-positive community youth researchers and professionals for preliminary analysis. Based on emerging themes, commonalities and major differences, a preliminary coding framework was developed [50]. Data were coded by two youth community researchers using Nud*istqualitative data analysis software [51]. After coding the first 10 transcripts, issues with the coding scheme were brought back to the larger stakeholder group and the scheme was refined and subsequently applied to the remaining transcripts.

Coded data were returned to the larger team for analysis. Members of the team were asked to fill out a work sheet for each code asking:

1. What was the range of experience here? What are the different ways that youth talked about their experience?
2. What are the general patterns that emerged? Generally how would you summarize what most young people had to say?
3. Which one or two quotes best summarize what you see here?

Weekly meetings were held to go over worksheets and discuss main themes, relevance and implications for each code. Collectively, the team's notes were discussed and summary tables constructed to capture the most common themes, gaps and issues.

## Results

Five main themes were identified with respect to the youth's use of and interest in the Internet as a health promotion strategy. These include: (1) high rates of Internet use and access; (2) issues around public and private terminals; (3) their use of the Internet primarily for communication and entertainment; (4) the rarity of health information seeking behavior in this group; and (5) wanting "one-stop shopping" from an e-health site.

### High Rates of Internet Use and Access

*"I'm online like all the time." – young man*

All of the youth we interviewed had used the Internet. Thirty four percent reported being online daily, 37% weekly and 29% said they were online monthly or occasionally. In addition, nearly half the youth we spoke with used instant messaging programs and two-thirds of the youth documented that they used e-mail at least once per week (Table 2).

**Table 2:** Frequency of technology use

**Technology Use**	**Web**	**Instant Messaging**	**Email**
Daily	12 (34%)	8 (23%)	13 (37%)
Weekly	13 (37%)	9 (26%)	10 (29%)
Occasionally or monthly	10 (29%)	10 (29%)	9 (26%)
Never	0 (0%)	8 (23%)	3 (9%)

**Figure 1:**
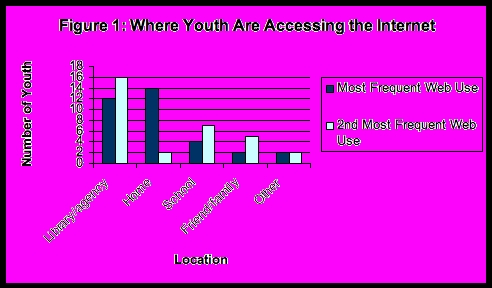
Where youth are accessing the Internet

These young people logged on from a wide range of public and private locations (Figure 1). Community centers, youth-serving organizations, AIDS-serving organizations, shelters, and public libraries were important points of Internet access both among housed and street-involved youth. Those who were actively street involved discussed the importance of using the Internet, particularly e-mail, as a vital communication mechanism. Nearly all the youth with a history of street involvement cited being online daily or weekly. While the quality of access (in terms of speed, privacy and freedom to surf) varied across locations, most youth were active users of public Internet terminals and access points.

Youth who documented lower rates of Internet use did not see access as the major barrier. Rather, these young people simply preferred other modes of communication and/or gathering information. As one young woman stated: "Ah, well honestly, this is nothing to do with HIV, but I rarely use it... I don't really have the patience for the Internet. I only use it on real necessity." She preferred using the telephone for communication and reading books for gathering information.

### Issues Around Public and Private Terminals

For many youth, private Web access was not a reality. Many were street-involved, some lived in subsidized housing and others lived in low income environments where Web access was not available at home. Thus, youth primarily talked about accessing the Web from public locations (Table 3).

**Table 3:** Public and private access

**Benefits of public access** Free access Anonymous Freedom	*"I wouldn't care, because Internet is used for so many things, it could be research for school..."* *"So many people use it -- they don't know who went on their sites. It doesn't matter. You go to Web station and it's the same thing, like, a thousand people might touch the same keys as you so..."* *"It's nobody's business anyway, you know. Like... why are you talking to me? I don't even know your name. Why would I care what you have to say, you know?"* *"I don't care where I go online. Nothin' really bothers me like that."*
**Drawbacks of Public Access** No real privacy Don't want to be publicly 'outted' as HIV positive Fear of emotional responses and having to 'deal' with others Usernames limit confidentiality	*"Never checked out health sites... Because usually when I go... either in a library or a cyber cafe I don't want people seeing what I'm looking up, just in case they take it the wrong way." The thing that would make him feel safe is an area where no one can see.* *"I don't like doing things in a library other than like check out books or something like that. People look over your shoulder. Or you're sitting--the computers are like right next to each other, and it's like--I've always felt uncomfortable even researching certain books, looking at a certain book. It's like can you see the title I'm typing in, don't look. You know I don't want, like, you to know, even if people aren't that nosy, I just don't want to risk it."* *"Really worried about checking out health info online... In the library I was always looking over my shoulder...What can you do, right? Somebody tell you - what's wrong, what's wrong' and you can't really say and you want to lash out so you have to leave."* *"Due to the fact that I don't have a computer at home and I use my computer from school. I have to sign in every time I use the computer and accessing sites like that leaves traces, so it leaves information behind... and I don't like that."*
**"Private" Access** better, but not 'totally' safe	*"I use the Internet from home, so I don't worry about stuff like that."* *"No. From a public in--no. Always my private. Because public, if you're going to a public library somewhere else, a little sign will pop up, 'information that you use or may enter may be seen by other people within this facility'. I don't like that. On my computer at least it will be like may be seen by people over the other--on the Internet and it's just like okay, I don't really like that either, there's not really big much of a difference, but at least it's a lot better when you're in the privacy and nobody can really pinpoint you..."*

Youth had mixed feelings about public access. Some saw the public portals as having an added sense of security and anonymity. In terminals where usernames were not required, they could feel free to browse the Web and search for confidential information without fear that they would be 'tracked down,' 'discovered' or 'outted' as being HIV-positive. They felt that because so many people used public access terminals all the time, their information or 'log histories' would be lost in the mix. One young person that had Internet access at home described searching for sensitive information at the library so her dad would not find out.

By contrast, other young people complained about the lack of privacy in public terminals. In particular, in libraries or shelters where computers were close together or peer networks were close-by, searching for sensitive or confidential information was not considered a possibility. Many youth were extremely leery about issues of confidentiality and were afraid that if they searched for information about HIV in public, others would find out about their status. Youth who needed special usernames or ID codes to access public terminals (e.g., at school) were extremely reluctant to search for confidential information.

Generally, youth that had home access often felt safer using private Internet access points than public ones. However, some youth acknowledged that even in the "privacy" of their own homes – they were not totally 'safe.' These youth were worried that their parents, friends or siblings might be able to trace their 'online movements.' Others worried that through the use of 'cookies' and other new technologies, others might be able to find out confidential information about them. As such, even 'safer' spaces were not seen as completely 'safe' or 'private.'

### Youth Use the Internet for Communication and Entertainment

*"I use it for everything and anything you can possibly think about in the world, and some things I'm not going to mention over tape." – young man*

Overwhelmingly, these young people used the Internet for communication (chat, message boards, e-mail, instant messaging). They talked at great length about their love of these Internet communication tools. Many of them had multiple e-mail addresses, and some talked about having multiple Internet identities.

These youth also spent a good deal of time surfing the Internet for entertainment purposes (e.g., games, music, sports, movies, pornography). For many, the Internet was seen as 'something to do' or a good alternative to television. Many of the young men mentioned interactive gaming. Generally, the Internet was seen as a way to have fun. As one young man put it, "I guess, like, the Internet for me is just like a time for playing games and chatting on the net."

These youth also documented using the Internet to search for information for school and work. Many were savvy Internet users and were able to describe complex search strategies for finding the information that they were looking for. Despite being sophisticated Internet users, few used to Internet to seek out health information.

### Health Information Seeking Behavior is Rare

*"Cause half the time I don't really know I have HIV because I don't think about it, 'cause it's not like something you really think about 'cause I'm doing well." – young woman*

Youth rarely talked about the Internet as a place where they sought health information (Table 4). A few young people described using the Internet regularly to learn about HIV, treatment options and community resources. These youth were 'expert' searchers; they were online regularly and knew how to access the information they were looking for. One young man in particular, was on HIV peer support sites regularly and saw his 'virtual friends' as important sources of social support and health information. However, these youth were in the minority.

**Table 4:** Searching for health information

**Many youth do not use the Internet for health information**	
Too early in diagnosis	*"I really haven't checked that stuff out yet."*
The Internet is a place to 'escape'	*"Because it is not interesting to me."* *"I guess, like, the Internet for me is just like a time for playing games and chatting on the net."*
Confidentiality	*"Because usually when I go to either a library or a cyber café, I don't want people seeing what I'm looking up, just in case they take it the wrong way."*
Prefers other methods of getting information (e.g., talking to health care providers, books)	*"Well, once in a while but hardly ever because I go to [Special school for street involved youth] and there's like a healthcare place you can go to so, if anything, I just go there if I have questions..."*
Doesn't like the Internet or computers	*"I don't really have the patience for the Internet...I just can't stand looking at computer screens, using the mouse, it feels so awkward so I don't like it."*
Doesn't know how	*"How do you find sites?"*
**Some youth have limited experience using the Internet for health information**	
A first stop for information	*"Yeah, when I first found out I had it, I went on sites, a few sites to find out what I wanted to do. I wanted to read up on some of it. Couldn't believe it, did a lot of crying the first few months. It was all so overwhelming."* *"I did like a couple of times like when I first found out but now it's just, I try not to think about it and try not to read much about it. Everything else is basically for adults."*
Some needed help negotiating information (friends, parents)	*"I did one time, yeah. It was helpful...Yeah. I had two of my other friends look with me, ahm, health information like different medications, how to take care of yourself, things like that, and I got a whole bunch of information on it."* *"When I first was diagnosed, I checked stuff out on the Websites. And then just all kinds of stuff...It was helpful because my dad was there. If I had been by myself, I probably wouldn't have understood anything. That's why you need to direct stuff towards teens."*
An available resource	*"I have been doing a lot of it recently, but I don't know, I'm still trying to take it all in. It's all like, thinking. I go to the site, I read it, like I don't know, a paragraph or two and I get psyched out of it, like, okay, I don't want to think about it, I don't want to think about it and I go off and play a new game. Then I go back to the site and read the next paragraph and then click off of it."*
**A small minority were expert searchers**	
Online daily, getting peer support	*"I always check this site out and there's a lot of people with HIV on there too, around the world. And I ask them questions... . (There's) Questions and answers, side effects on drugs, I mean, hundred medications, the whole nine yards."*
Savvy searchers	*"You just search google or yahoo and tons of stuff comes up."*

When probed about why most did not use the Internet to access health information, they had a variety of responses. For some, the Internet was a place to 'escape' to. They saw the Internet as being primarily about entertainment (e.g., "I use the Internet to play card games and interact with other people.") For others, seeking health information was not seen as a priority because HIV was a relatively small part of their identity. As one young man explained, "It is, after all, only three letters." A small subset of youth worried that if they searched for health information about HIV online, someone (their ISP provider or others around) might find out about their HIV status. One young woman had adopted strategies for managing these issues, "[at the agency] the computers are so close and there are a lot of people I know there...but at the library I feel safer." Other reasons that youth provided included: preferring other methods of getting information; hating computers; not knowing how to access appropriate information; and not being ready yet to find out more information (i.e., too early after diagnosis).

Other youth who had experimented with using the Internet for health information complained that: a) there was too much out there and it was hard to prioritize and figure out "what's what"; b) most of the information that was out there was unintelligible and c) they found the experience somewhat overwhelming. Some adopted strategies of asking friends or family to search with them and act as translators or interpreters. As one young man described, "It was helpful because my dad was there. If I had been by myself, I probably wouldn't have understood anything. That's why you need to direct stuff towards teens."

### Youth Want "One-Stop Shopping" From an eHealth Site

*"Like basically, one-stop information location for positive youth. Like everything and anything you can put in there, but put it into a format that youth can understand, right? Something to create and have some fun with!" – young man*

Despite the Internet's rare use for health information, when asked if they would visit a Website specifically designed by and for HIV-positive youth, most of the young people we interviewed were extremely enthusiastic about the possibilities of the Internet for health promotion. Nine percent said they would use a site specifically developed for positive youth everyday. Twenty-nine percent said they would use it regularly, 43% said they would use it once in awhile and 20% said they would never use such a site. Youth in the "once in awhile" category were generally enthusiastic about the concept. While they did not see their HIV status as being a major part of their identity, they were nevertheless interested in being able to access relevant content when appropriate (e.g., when they had specific questions). The minority of youth that would not access these resources gave the following reasons. They were either: (a) not interested in HIV health information resources generally; (b) unenthusiastic about the Web; or (c) concerned about privacy.

**Table 5:** What youth want from an eHealth site

**Social Support & Communication Opportunities**	
Chat Rooms	*"Chat rooms where people can chat about how they're feeling, how they're doing... they can write to each other and stay in contact..."*
Message Boards	*"Message boards... for people to connect with each other - you feel so alone, you want to talk about stuff and share ideas... you can post your feelings, a poem or something..."*
**Information**	
Treatment	*"Information about medication, information about other options out there, information about doctors that are youth oriented or they are good with you, so basically an investigation on doctors."*
Resources	*"Like you know, there's a whole bunch of agencies or services that you have to know. We all don't know of things that are out there."*
HIV/AIDS	*"Like about how you get it and stuff."*
Harm reduction	*"Like how to make sure you don't spread it and stuff."*
'Health' generally	*"How to maintain your health and be good to your body."*
**Interactive Components**	
Games, quizzes	*"Funkiness, coolness... make it fun and comfortable."*
Q & A	*"Like ask the doctor."*
**Privacy Protected**	
Nicknames	*"If you don't want to use your real name, you should be able to use a code."*
Password	*"Password is a big thing."*
Confidentiality Agreements	*"[You should have] a privacy or confidentiality agreements... so youth will understand that their information is not going to go out to somebody else on the net."*
Discreet Look	*"Why do all you guys use big, red letters? Big- big red bubble letters [that say HIV] all over the site!?"*
Protection from prowlers	*"It might not be a good idea because the older guys might go in there and start going after the youth. And it might be a really uncomfortable situation for those youth."*

Youth were extremely specific about what they wanted from e-health strategies targeted towards them (Table 5). First and foremost, young people wanted an opportunity to share and connect with each other; chats and message boards were seen as the main attraction to heading towards a site specifically for HIV-positive youth. Nearly every young person we spoke with recommended the creation of a chat space. Many youth described feelings of isolation and loneliness and felt that the opportunity to connect with other youth in similar circumstances could be extremely valuable. They talked about the importance of connection and prioritized chats and message boards over other possible Internet information applications (e.g., didactic information). Many of them worried, however, about "prowlers" and sexual solicitations in sites that were geared towards youth audiences and were concerned about how those might be managed.

Youth embraced the concept of "one-stop shopping" or one site that would be able to answer all their questions. Many talked with frustration about how so many sites 'out there' that dealt with HIV were not 'youth-friendly' or 'user-friendly' and were hard to understand. They did not enjoy getting 'lost' in complicated links.

Study participants wanted the look and feel of a site geared towards them to be discreet and not 'obviously' about HIV so that they could access it in public forums. In order to protect their privacy, youth did not want to be asked for their real names. They were happy to provide nicknames and felt that passwords provided added protection. Also, they wanted to see privacy or confidentiality agreements that assured them that their confidentiality and anonymity would not be compromised.

## Discussion

Our study found that HIV-positive youth are online and are Web savvy users. A surprising finding was that the youth who were perhaps most impoverished and marginalized (those who were either currently or had a history of being street involved) demonstrated high rates of Internet use. This is in sharp contrast to American findings of adult populations living with HIV [41,42].

This study supports evidence from investigations of adults living with HIV that communication is a primary reason that people living with HIV use the Internet. However, unlike adults, our study did not identify advocacy as something that youth did online. Information seeking was secondary [38,39].

Although they go online with regularity, the HIV-positive youth in our study rarely searched for health information. This was due to personal and institutional barriers including: lack of interest; difficulty assimilating information geared towards adults; fear of disclosure; and inadequate private access. Our findings that youth living with HIV use the Internet primarily for communication and entertainment is consistent with other studies that have looked at Internet use among more general youth populations [52]. In addition, the problems documented around quality of access are also found among other youth populations [53].

Results of a national needs assessment conducted by the Canadian AIDS Society in 2000 concluded that, "Nationally...there was a huge lack of services for HIV-positive youth. These missing services ranged from support groups, to accessible treatment information, and basic living necessities for positive youth." [6] Similarly, Toronto's Positive Youth Outreach 2000 Survey found that youth ranked treatment information amongst their top six most pressing needs [18]. Furthermore, they cited the Internet as one of the best ways to give them information. Identifying a need for further venues of social support, these youth documented that Chatrooms/ Listserves, phone lines and social events were the top three services they wanted. This study confirmed that, indeed, the Internet may be a viable way to impart health and treatment information, provided the content, look and feel of such materials were created and presented in 'youth friendly' formats.

One limitation of this study is that we recruited young people from youth- and AIDS-serving organizations and health care settings. As such, the youth we spoke to were generally well connected to health and/or social services, which may have provided them higher rates of public Web access. The qualitative nature of our study also makes it difficult to generalize our results to all HIV-positive youth.

Nevertheless, this study suggests that if content were developed specifically for HIV-positive youth and marketed to them, they would be interested. Targeting young HIV-positives for health promotion messaging may be both feasible and desirable [54]. Given the success of computerized and online health promotion strategies with other populations, this may prove to be an important health promotion strategy.

Health care providers should be aware of the need for providing information to HIV positive youth in non-traditional formats. Health care providers may want to familiarize themselves with youth-friendly resources that are already available (e.g., http://www.livepositive.ca or http://www.youthhiv.org/). Referring youth clients to appropriate Web sources may be an important additional tool for health care providers and health promoters for supplementing 'regular care'. Finally, the onus is on e-health developers to better understand the needs of this vulnerable population and continue to expand and create appropriate, relevant and up-to-date content.

## Appendix 1

### Interview Guide

**Goals:** How can youth living with HIV be supported in achieving their goals?

- What are your hopes, goals, and aspirations for the future?
- What might help you to achieve your goals? What could you do for yourself?
- Have your goals changed as a result of learning your HIV status? How?
- What kinds of programs do you wish were around for young people like you?

**Treatment:** How can youth living with HIV be supported in making treatment or self-care decisions?

- How prepared do you feel to take care of your health?
- What kinds of problems do you face when taking care of your health?
- Do you feel like you have choices about treatment? Do you know what your choices are?
- What kind of information would you like on different treatment options for young people living with HIV/AIDS?
- What are some of the 'health topics' that you feel like learning more about?
- How can you be supported in making treatment or self-care decisions?
- What advice would you have for other HIV positive youth that are trying to make treatment decisions?

**Support:** How can youth living with HIV be supported?

- Where do you feel comfortable going for health information?
- Who do you turn to for moral/social support?
- How open are you about your HIV status with people in your life?
- Do you ever feel lonely or excluded because of your HIV status?
- Even though you are a diverse group of individuals - what common experiences do you think HIV+ youth might share?

**Internet:** How do youth living with HIV use the Internet?

- Do you ever use the Internet? What for? (Do you ever use it to get health info?)
- Do you know about any sites are out there for HIV+ youth? Do you use them? For what? Do you feel safe using them? Why/why not?
- If we were going to create a new Website for HIV positive youth in Canada - what sorts of things should we be sure to include? (What content/information would you like to see on the site? e.g. counselors, chat rooms, information)
- What would make you feel safe accessing it? Do you have a safe place to access it from? What sorts of things could we do to protect your privacy? (Would you feel safer accessing the site if it had a privacy/password age before you get to the homepage?)

## Appendix 2

### Coding Scheme

1. **Relationships**
  1. **To parents and family**
    1. Loving and supportive
    2. Antagonistic
    3. Abusive, dysfunctional
    4. Other
  2. **To health care providers**
    1. Faith - they know best
    2. Frustration
    3. Other
  3. **To other youth**
    1. Friends
    2. Other youth generally
    3. To other youth with HIV
    4. Other
  4. **To partners, lovers (boyfriend/girlfriend)**
  5. **To other institutions**
    1. Church & Spirituality
    2. ASO's
    3. Youth Orgs
    4. Youth Shelters
    5. Counsellors, Case workers
    6. Hospital clinics
    7. School
    8. Kids Help Phone
    9. Other
2. **Isolation, loneliness**
3. **Future**
  1. Living day by day (not future oriented)
  2. Uncertainty (in general)
  3. Long term vs. short term
  4. About health
  5. About relationships, partners
  6. About family of origin
  7. About starting own family
  8. About school
  9. About career, employment
  10. About financial stability
  11. About housing
4. **Goals changed**
  1. Yes - why
  2. No - why
5. **Feelings about HIV**
  1. I'm a normal kid - no big deal
  2. Regrets, Guilt
  3. Grief
  4. Shame - HIV as "dirty" or "foreign" or "bad"
  5. Angry, resentful
  6. Small part because I am overwhelmed with everything else
  7. Just is a small part of me
  8. "death sentence", feeling mortal
  9. conspiracy theories
  10. Stigma
  11. Acceptance or integration of HIV in life
  12. Other
6. **Life in shelters/Street Issues**
  1. Prostitution
  2. Drug use
  3. Abuse
  4. Hygiene
  5. Feelings about living in shelters
  6. Rules
  7. Panhandling
  8. Respect
  9. Other
7. **Issues around disclosure**
  1. Confidentiality
  2. Bad experiences/Stigma/Discrimination
  3. Good experiences
  4. Why should I tell you? (I wouldn't if I had a growth on my foot)
  5. Other
8. **Advice for other youth**
  1. Stay positive
  2. Think before you act
  3. Find people you trust
  4. Other
9. **Information**
  1. Want to know more about..
  2. Fear of finding out more...
  3. Lack of interest...
  4. Saturated (savvy about what is out there, accessing services)
  5. Where I go to get info
10. **Other health issues**
  1. Eating disorders
  2. Hygiene
  3. Vitamins, nutrition
  4. Addictions & substance use
  5. Violence/abuse
  6. Mental health issues
  7. Asthma
  8. STDs
  9. Cancer
  10. SARS
  11. Other
11. **Self-care stuff**
  1. Diet, vitamins, nutrition
  2. Exercise
  3. Sleep
  4. Choosing healthy, supportive relationships
  5. Taking care of yourself is tiring, exhausting, hard work
  6. Getting help
  7. Apathetic around self-care
  8. Alternative therapy (e.g., acupuncture)
  9. Other
12. **Knowledge, Attitudes & Behavior Around Medication**
  1. Why take it?
  2. Knowledgeable/Informed
  3. Have no choice
  4. Side effects & concerns
  5. Taking medicine as a young person
  6. Not ready for it
  7. Barriers to accessing meds
  8. Figuring out what is credible
  9. Avoiding it
  10. Adherence, interruption
  11. Other
13. **Coping Strategies**
14. **Level of Internet Usage, what people do (and don't do) online**
  1. Chat/communicate
  2. Entertainment
  3. Work (job or school)
  4. Health info
15. **Ideas for Website**
16. **Privacy, Safety and the Internet**
  1. Public Access Good
  2. Public Access Bad
  3. Private access
  4. Presentation of site
  5. Passwords and confidentiality
  6. Protection from prowlers
  7. The Internet as anonymous, safe
  8. The Internet as unsafe
